# Teratogenic Effects of Alcohol on Brain and Behavior

**Published:** 2001

**Authors:** Sarah N. Mattson, Amy M. Schoenfeld, Edward P. Riley

**Affiliations:** Sarah N. Mattson, Ph.D., is an assistant professor of psychology and associate director of the Center for Behavioral Teratology, San Diego State University (SDSU), San Diego, California. Amy M. Schoenfeld is a graduate student in the SDSU/University of California-San Diego Joint Doctoral Program in Clinical Psychology, San Diego, California. Edward P. Riley, Ph.D., is a professor of psychology and director of the Center for Behavioral Teratology, San Diego State University, San Diego, California

**Keywords:** fetal alcohol syndrome, prenatal alcohol exposure, teratogenesis, brain imaging, neuropsychological assessment, cognitive and memory disorder, basal ganglia, corpus callosum, cerebellum, hippocampus, electroencephalography, magnetic resonance imaging, positron emission tomography, single photon emission computed tomography

## Abstract

Children prenatally exposed to alcohol can suffer from serious cognitive deficits and behavioral problems as well as from alcohol-related changes in brain structure. Neuropsychological studies have identified deficits in learning and memory as well as in executive functioning both in children with fetal alcohol syndrome and in children with less severe impairments. Both groups of children also exhibit problem behaviors, such as alcohol and drug use, hyperactivity, impulsivity, and poor socialization and communication skills. Brain imaging studies have identified structural changes in various brain regions of these children—including the basal ganglia, corpus callosum, cerebellum, and hippocampus—that may account for the cognitive deficits. Functional brain imaging studies also have detected changes in alcohol-exposed children indicative of deficits in information processing and memory tasks.

Prenatal alcohol exposure can have serious and permanent adverse effects on children. The extent and severity of a child’s condition depends on several factors, such as how much alcohol the pregnant mother consumed and how often and at what point during her pregnancy she drank. The most serious outcome is fetal alcohol syndrome (FAS), the diagnosis of which is based on three criteria: (1) growth deficiency manifested by small overall height and small head size (i.e., microcephaly); (2) central nervous system disorders; and (3) a distinctive pattern of abnormal facial features. Other children with histories of heavy prenatal alcohol exposure, however, often do not meet the diagnostic criteria of FAS. These children, who typically lack the characteristic facial features of FAS, have variously been labeled as having fetal alcohol effects (FAE), alcohol-related neurodevelopmental disorder (ARND), or prenatal exposure to alcohol (PEA). Both children with FAS and those with related disorders can be born to women known to drink in a heavy episodic fashion or more regularly during pregnancy. For the remainder of this article, children with histories of prenatal alcohol exposure who do not meet the diagnostic criteria of FAS are referred to as either having FAE or PEA. When available, data from such children are noted; otherwise, the results presented in this article refer to children diagnosed with FAS.

Children with histories of heavy prenatal alcohol exposure show evidence of changes in brain structure and function as well as a variety of behavioral effects presumably resulting from this insult to the brain. Most of the research conducted among alcohol-exposed children and adolescents has focused on either the structural or behavioral effects. Only recently have studies begun to demonstrate the relationship between the two areas—that changes in brain structure could negatively affect behavior. This article summarizes the results of neuropsychological studies analyzing alcohol’s teratogenic (i.e., damaging to the developing fetus) effects on behavior and of brain imaging studies analyzing alcohol’s effects on brain structure. It then highlights the existing connections between those two areas of research. For more extensive coverage of these topics, the reader is referred to review articles by Mattson and Riley (1998) and Roebuck and colleagues (1998).

## Results from Neuropsychological Studies

Generally, heavy prenatal alcohol exposure is associated with deficits in a wide range of areas of function, including both cognitive functioning (e.g., general intellectual functioning, learning of new verbal information, and performance on visual-spatial tasks) and fine- and gross-motor performance. Neuropsychological studies have analyzed the cognitive impairment of children with histories of prenatal alcohol exposure. Although many of these studies have focused on children diagnosed with FAS, several analyses have included children with FAE or PEA.

Importantly, many studies show that strong similarities exist between children with FAS and children with FAE/PEA. For example, studies of overall cognitive ability in FAS children typically report average IQ scores in the borderline range of functioning (i.e., in the low 70s), although they can range from “intellectually deficient” (IQ scores less than 70) to “average” (IQ scores between 90 and 109). Children with FAE or PEA also show deficits in IQ scores, although these deficits typically are not as severe as in the children with FAS (Streissguth et al. 1991; Mattson et al. 1997).

In addition to overall intellectual or cognitive deficits, researchers have evaluated a broad range of cognitive functioning areas in children with FAS, FAE, or PEA, including language skills, visual-spatial functioning, fine-motor behavior, nonverbal learning, and academic performance. In general, alcohol-exposed children both with and without FAS show significant impairments in all neuropsychological areas with few qualitative differences observed between the FAS and PEA/FAE groups. Similarly, high levels of prenatal alcohol exposure are related to an increased risk for cognitive deficits across a range of functioning areas, which again can occur in children both with and without a diagnosis of FAS.

### Learning and Memory

Both anecdotal information and results from animal studies have indicated that prenatal alcohol exposure can affect learning and memory. Studies of children with FAS generally have supported this observation, although the deficits in memory may not be as global as was once thought. For example, one study investigated verbal learning and memory in children with FAS and in non-alcohol-exposed control children (Mattson et al. 1996*b*). The study found that although the FAS children demonstrated some deficits in memorizing verbal information, these deficits resulted from difficulties with the acquisition of the information rather than with the ability to remember the information over time. Other studies also have revealed similar deficits in the acquisition of nonverbal information in alcohol-exposed children (Mattson and Roebuck in press), suggesting that learning deficits occur in both verbal and nonverbal arenas and are likely to cause significant impairment in diverse areas of functioning. It is unclear, however, whether the degree of impairment for each child differs between the verbal and nonverbal areas of function.

Some studies suggest that children with FAS can perform well when memory function is tested in a different way, for example in tests of implicit memory—a type of memory that is not under conscious control. When subjects successfully perform implicit memory tests, they may use information from previous tasks without being aware that they have done so. In one study, investigators showed children with FAS lists of words and asked the children to rate those words on likeability (Mattson and Riley 1999). (This rating component served to enhance the children’s attention to the words.) Later in the testing session, the children were asked to complete partial words (e.g., MO_____ or SM_____) with the first word that came to mind (e.g., MOUSE or SMILE). The children were not reminded of the previous words nor prompted to remember them by the examiner. Nevertheless, both FAS and control children were more likely to complete the partial words with words from the previous task than with new words. These results indicated that both groups of children used implicit memory and that prior exposure helped them learn and memorize the words.

Taken together, these findings suggest that although children with FAS may have significant impairments in learning new information, their overall memory function is complex and may not be as globally affected as was commonly thought. Nevertheless, specific aspects of memory may be affected by prenatal alcohol exposure.

### Executive Functioning

The term “executive functioning” refers to a group of higher-level cognitive abilities, such as solving problems, thinking abstractly, planning ahead, and being flexible in one’s thought processes. These types of skills are independent of overall intellectual function and influence whether and in what manner a person can complete a task. Conversely, tests of other cognitive abilities tend to assess how well, or at what level, a person performs a skill (Lezak 1995). (For more information on executive functioning and the effects of prenatal alcohol exposure on these skills, see the article in this issue by Kodituwakkuand colleagues, pp. 192–198.)

Children with heavy prenatal alcohol exposure (both with and without FAS) have demonstrated impairments on executive functioning tasks (Kodituwakku et al. 1995; Mattson et al. 1999). Importantly, in these studies the children’s deficits in executive function were unrelated to their overall intellectual levels. This finding is supported by a recent study among adults with FAS or FAE, which found that the subjects’ deficits in executive functioning were greater than would have been predicted if they were related to overall IQ scores (Connor et al. in press).

Deficits in executive functioning can have real-life implications for people prenatally exposed to alcohol. For example, people with heavy prenatal alcohol exposure may act without first considering the consequences of their behavior or they may have difficulties with activities that require problem solving or with planning a sequence of activities. These types of deficits may explain why children with heavy prenatal alcohol exposure, even those with average IQ scores, have difficulty succeeding in school.

### Psychosocial Deficits and Problem Behaviors

Studies involving parent reports and interviews have suggested that alcohol-exposed children with or without FAS not only have cognitive deficits but also are at high risk for problem behaviors that can interfere with their participation in home, school, and social environments. For example, these children appear to be at increased risk for psychiatric disorders, trouble with the law, alcohol and other drug abuse, and other maladaptive behaviors (Streissguth et al. 1996). Moreover, they are more likely than non-alcohol-exposed children to be rated as hyperactive, disruptive, impulsive, or delinquent (Roebuck et al. 1999; Mattson and Riley 2000). Similarly, on measures of adaptive ability and skills necessary to perform age-appropriate daily living activities, adolescents and adults with FAS often exhibit poor socialization and communication skills. In addition, the majority of these adolescents and adults display significant maladaptive behaviors (e.g., impulsivity) and are less likely to be living independently (Streissguth et al. 1991; Thomas et al. 1998). It is noteworthy that these problems occur in people prenatally exposed to alcohol whether or not they meet the criteria of FAS and occur to a greater extent than would be predicted by the person’s general intellectual functioning or demographic factors.

## Results from Brain Imaging Studies

The neuropsychological and behavioral deficits described in the previous section represent real-life manifestations of the effects of prenatal alcohol exposure. Although deficits on these measures are thought to provide evidence of underlying changes in brain structure or function, they represent only indirect measures of such brain changes. Alcohol’s direct effects on brain development were already noted in the earliest reports of FAS (Jones et al. 1973), however, and autopsy studies of brains from people with FAS noted numerous and widespread brain abnormalities. Because these cases represented only the most severely affected children, it is problematic to generalize the findings to all people living with FAS. With the advent of numerous structural imaging techniques, such as magnetic resonance imaging (MRI), and functional imaging techniques, such as electroencephalography (EEG), positron emission tomography (PET), and single photon emission computed tomography (SPECT), however, researchers can now study the living brains of alcohol-affected children in a relatively noninvasive fashion.

### Structural Brain Imaging

**Figure 1:**
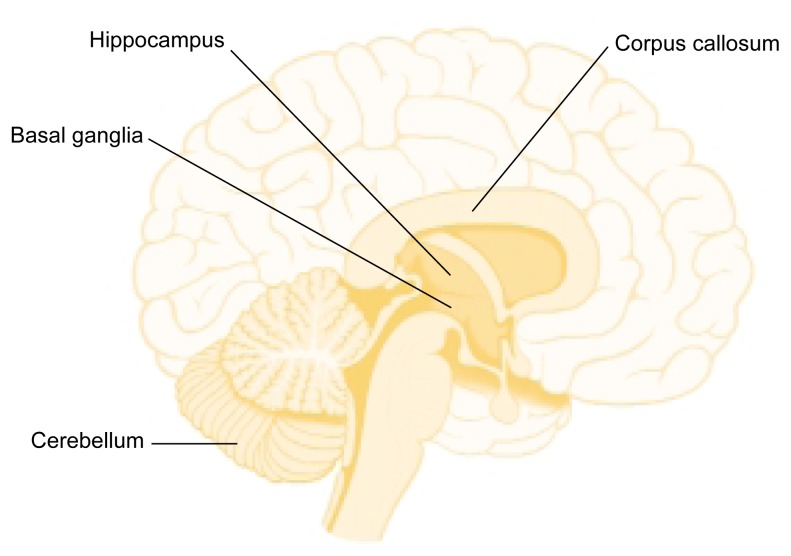
Brain areas affected by prenatal alcohol exposure.

Imaging studies using MRI have revealed several differences between the brains of alcohol-exposed and non-exposed individuals. Consistent with the characteristic small head size, which is one of the diagnostic criteria for FAS, imaging studies show a decrease in the overall size of the brain of FAS children (Roebuck et al. 1998). To determine whether this size reduction results from global and diffuse alcohol effects on all brain areas or is limited to specific regions, researchers have assessed specific structures in proportion to overall brain size. This approach can determine whether specific, disproportionate reductions occur in some brain areas. These investigations have focused on several brain areas, including the basal ganglia, corpus callosum, cerebellum, and hippocampus (see figure 1).

#### Basal Ganglia

The basal ganglia are a group of nerve cell clusters (i.e., nuclei), including the caudate nucleus, putamen, and globus pallidus. They are involved in motor abilities and cognitive functions, such as the executive functions described earlier. MRI studies have revealed that the basal ganglia are affected by heavy prenatal alcohol exposure and are disproportionately reduced in volume in children with FAS and PEA. More detailed examination of the components of the basal ganglia found that the reductions are not uniform and that the caudate nucleus appears to account for most of the size reduction in the basal ganglia (Mattson et al. 1996*a*; Archibald et al. 2001).

The caudate nucleus is the portion of the basal ganglia involved in cognitive functions. For example, skills such as the ability to shift from one task to another, inhibition of inappropriate behavior, and spatial memory, which are impaired in people with prenatal alcohol exposure, have been related to the basal ganglia in other populations, such as patients with Huntington’s disease (Mattson et al. 1996*a*; Mattson and Riley 1999; Archibald et al. 2001). Accordingly, it is possible that the reductions in the caudate nucleus account for some of the cognitive deficits seen in people with prenatal alcohol exposure. This hypothesis is particularly appealing because the caudate nucleus also has extensive neural connections to the frontal lobes of the brain, which traditionally are thought to mediate higher cognitive and executive functions.

#### Corpus Callosum

The corpus callosum is a large bundle of nerve fibers connecting the two hemispheres of the brain, thereby allowing the left and right sides of the brain to communicate with one another. Corpus callosum abnormalities have been linked to deficits in attention, intellectual functioning, reading, learning, verbal memory, and executive and psychosocial functioning, all of which are impaired in alcohol-exposed people. MRI studies and autopsy reports suggest a vulnerability of the corpus callosum to prenatal alcohol exposure; such studies found that people with FAS exhibit abnormalities ranging from a thinning to complete absence (i.e., agenesis) of the corpus callosum (Roebuck et al. 1998). When specific regions of the corpus callosum were analyzed, researchers found that the front-most area—the genu—and the back-most areas—the isthmus and splenium—were disproportionately reduced in size (Riley et al. 1995). Moreover, the rate of agenesis of the corpus callosum may be higher in people with FAS than with any other developmental disorder (Jeret and Serur 1991; Riley et al. 1995).

**Figure 2:**
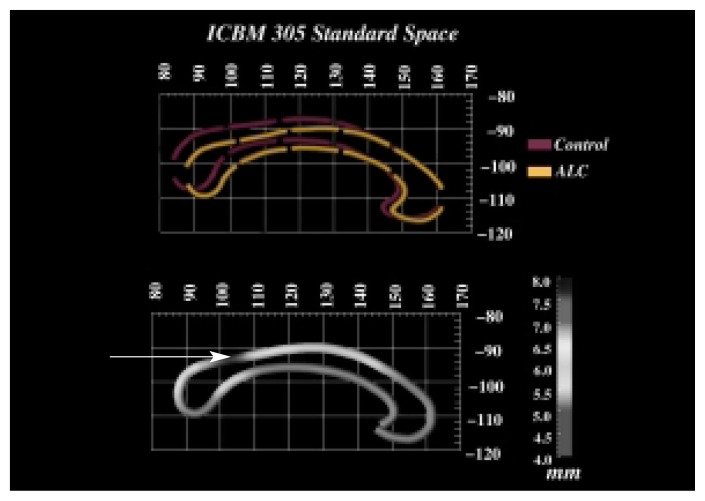
(Top) Average outlines of the corpus callosum (i.e., the bundle of nerve fibers connecting the brain’s right and left hemispheres) in alcohol-exposed subjects (ALC) and non-alcohol-exposed control subjects. The corpus callosum is oriented so that the front of the head is to the right and the back of the head is to the left. The figure shows that the corpus callosum of the ALC is displaced in three-dimensional space compared with that of the control subjects, with the greatest displacement occurring in the isthmus and splenium at the back of the corpus callosum. (Bottom) A map showing the average displacement in millimeters between the ALC and the control subjects. Darker area (see arrow) indicates greater displacement between the two groups. Greater displacement is associated with greater performance impairment in certain tasks. SOURCE: Figure courtesy of Dr. Elizabeth Sowell.

Recently, researchers analyzed in more detail the shape and location of the corpus callosum of FAS and PEA children as well as of control children (Sowell et al. 2001). The study not only confirmed that the corpus callosum was reduced in size, specifically in the splenium, but that it was also significantly displaced in three-dimensional space (see figure 2). After equalizing all brains for brain size and the location of other structures located along the midline of the brain, the average location of the corpus callosum for the alcohol-exposed children was compared with the average location for the control children. This analysis found that the corpus callosum in the alcohol-exposed children was displaced compared with the control children, with the biggest differences in the area of the isthmus and splenium, both of which are located in the back of the corpus callosum. Furthermore, this corpus callosum displacement was highly related to the children’s performance on a verbal learning task. In other words, children with greater displacement exhibited more substantial performance impairments.

#### Cerebellum

Another area of the brain that is affected by prenatal alcohol exposure is the cerebellum, which is involved in both motor and cognitive skills and is located at the base of the brain. For example, damage to the cerebellum has been implicated in learning deficits as well as in balance and coordination, all of which are impaired by prenatal alcohol exposure. A recent study found that the overall volume of the cerebellum was disproportionately reduced relative to overall brain size in people with FAS compared with control subjects (Archibald et al. 2001). These findings partially replicate previous reports of reduced cerebellar size in FAS and PEA children (Sowell et al. 1996). In addition to the overall reductions in the size of the cerebellum, studies conducted in both humans and animals suggest that a specific region of the cerebellum—the anterior portion of the cerebellar vermis—is particularly affected by alcohol exposure before or shortly after birth^1^ (Goodlett et al. 1990; Sowell et al. 1996).

#### Hippocampus

The hippocampus is a structure that lies deep within the temporal lobe of the brain and is involved in memory. Although the precise function of the hippocampus in specific aspects of memory is controversial, it probably plays a role in the consolidation of memories. For example, in adults with hippocampal damage, the most obvious effect is a loss of the ability to store new memories (i.e., anterograde amnesia). Animal studies have long suggested that this area is affected by prenatal alcohol exposure (Berman and Hannigan 2000). Moreover, an MRI study of children with FAS documented volume asymmetries in the hippocampus, with the absolute volume of the hippocampus in the left temporal lobe smaller than that of the corresponding area in the right temporal lobe (Riikonen et al. 1999). Although such differences also exist in adults with normal neurological function, the extent of the asymmetry was greater in the FAS children than in the control children. Conversely, another study found that the hippocampus was less affected than some other brain regions in FAS children (Archibald et al. 2001). In that study, the reduction in the volume of the hippocampus was proportionate to the reduction in overall brain size, whereas other brain areas showed greater reductions in volume.

Behavioral studies have supported the hypothesis that the hippocampus might be affected in children with prenatal alcohol exposure. For example, people with prenatal alcohol exposure have been reported to exhibit deficits in spatial memory as well as other memory functions associated with the hippocampus (Uecker and Nadel 1996). However, the memory deficits in alcohol-exposed children require more detailed study and should be integrated with information about the integrity of the hippocampus.

This issue also points out a limitation of structural imaging, namely that this approach only determines the size of a particular brain structure but does not indicate whether the structure is functioning correctly. To determine how a particular brain area functions under different conditions and whether these functions are altered by prenatal alcohol exposure, researchers are turning to functional brain imaging approaches, discussed in the following section.

### Functional Brain Imaging

Functional imaging techniques allow researchers to study how the brain works, either at rest or when presented with a task. Because some functional techniques are more invasive or technically difficult to conduct with children, only a small number of studies using these techniques have been conducted in FAS children. The most commonly used technique in these studies is electroencephalography (EEG).

#### EEG

The EEG measures the brain’s spontaneous electrical activity by recording signals from the brain with electrodes placed on the scalp. These signals can be visualized as waves with specific frequencies, such as alpha, beta, and theta waves. Early studies on infants suggested that EEG may be a sensitive measure of changes in brain function resulting from prenatal alcohol exposure (Ioffe and Chernick 1990). More recent studies of children and adolescents with FAS found that approximately one-half of these subjects had clinically suspect EEG readings (Kaneko et al. 1996*b*). Furthermore, subjects with FAS exhibited reductions in the power or strength of the alpha frequencies, which is the predominant type of activity when a person is relaxed. These reductions were seen predominantly in the left hemisphere and suggest immature brain activity.

Using similar techniques, it is possible to measure the brain’s electrical response to specific sensory stimuli (i.e., event-related potentials). These event-related potentials can be visualized as spikes in certain brain waves. One of these spikes is called P300, because it typically occurs approximately 300 milliseconds after the stimulus; it appears to reflect the cognitive aspects of information processing. Using EEG analyses, researchers found that the P300 spikes occur with a delay (i.e., have a prolonged latency) in a certain brain region, the parietal cortex, in FAS children (Kaneko et al. 1996*a*,*b*). This finding suggests that children with FAS may have deficits in information processing. Thus, electrophysiological measurements are powerful tools in the study of FAS; future studies combining them with localizing brain imaging may provide further information about brain function.

#### PET

The PET technique allows researchers to monitor the activity of specific brain regions by generating images of metabolic or physiologic processes, such as blood flow or breakdown of sugar molecules, in the tissue. For this approach, the subject is injected with small amounts of radioactive material so that brain activity in the region of interest can be measured while the subject performs a task. These tasks can range from the simple, such as moving a finger, to the complex, such as recalling information. One PET study assessed brain activity in adolescents and adults with FAS who showed no severe mental retardation (i.e., who were high functioning). The study revealed reduced metabolic activity in the caudate nucleus and in the thalamus when the subjects were at rest (Clark et al. 2000). These functional data support the structural data, such as the reduced size of the caudate nucleus, suggesting that subcortical brain regions may be especially sensitive to prenatal alcohol insult.

#### SPECT

The SPECT technique is similar to PET, and although it is less powerful, it is more commonly available. However, only one study of FAS children has used this technique. In that study, the investigators found that FAS children exhibited similar metabolic activity in both hemispheres of the brain (Riikonen et al. 1999). Normally developing children, in contrast, show greater resting activity in the left hemisphere than in the right hemisphere. These results are consistent with the EEG findings described above and may support verbal or language deficits in FAS children.

#### Functional Magnetic Resonance Imaging (fMRI)

The newest functional technique used to study activity in the living brain is fMRI. Its main advantage is that it is less invasive than PET or SPECT because it does not involve injecting the subject with radioactive substances; moreover, it is more commonly available. Similar to PET and SPECT, fMRI allows researchers to visualize brain activity while the subject performs a cognitive task. To date, no published reports exist of fMRI studies in people with prenatal alcohol exposure; however, such studies are currently underway. One preliminary report described an fMRI study of working memory—using information held in memory for a short period of time—in four adults with FAS or FAE (Connor and Mahurin 2001). The study revealed activation in an area called the dorsolateral prefrontal cortex in the FAS subjects but not in control subjects. This area is thought to play a role in higher cognitive functions, such as the executive functions described above. This result suggests that the working memory task was more difficult for the alcohol-exposed subjects and required greater involvement of this region of the frontal lobe compared with the control subjects.

## New Image Analysis Techniques

In addition to improvements in brain imaging techniques, new ways of analyzing the data obtained with these techniques are providing scientists with insights about the damaging effects of prenatal alcohol exposure. One of those techniques is called brain mapping. It uses a structural MRI analysis but provides greater visualization of all brain structures. As a result, researchers can study the whole brain at once, rather than focus on specific brain regions, and therefore can localize brain abnormalities more easily than with previous techniques.

**Figure 3:**
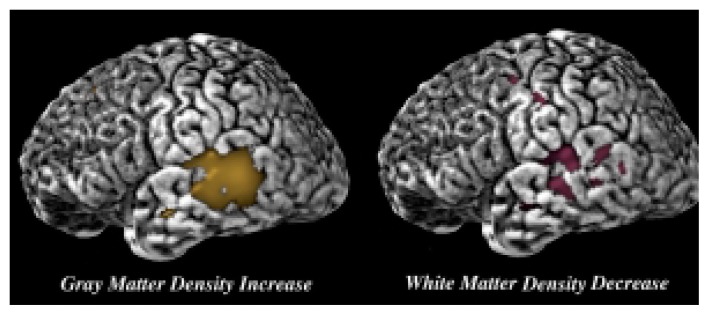
Changes in brain tissue density in children with heavy prenatal alcohol exposure. A representative brain is shown with the back of the brain facing the reader’s right. Brain-mapping studies detected areas of increased gray matter density (shown in yellow on the left) as well as areas of reduced white matter density (shown in red on the right) in the parietal lobe. SOURCE: Figure courtesy of Dr. Elizabeth Sowell.

Sowell and colleagues (2001*b*) have used the brain mapping technique to analyze and compare brain images of people with FAS or PEA and non-alcohol-exposed control subjects. Consistent with the results of Archibald and colleagues (2001), the study detected disproportionate reductions in the brain’s white matter, which contains the nerve cells’ extensions (i.e., axons) that connect nerve cells with each other. Conversely, the brain’s gray matter, which contains the nerve cell bodies, showed reductions that were not as great. In addition, the parietal lobe, which is involved in visual-spatial processing and in the integration of sensory information, appeared to be especially susceptible to alcohol’s effects. Thus, once overall brain size was accounted for, both the volume (Archibald et al. 2001) and the density (Sowell et al. 2001*b*) of white matter in this region were significantly reduced (see figure 3). Conversely, the gray matter density in the parietal cortex was significantly increased (Sowell et al. 2001*b*). These findings lend additional support to the suggestion that alcohol’s effect on the developing brain is not global in nature but, rather, affects specific brain regions selectively.

## Future Directions

The studies reviewed here provide clear evidence that both brain structure and brain function are affected by heavy prenatal alcohol exposure. More recent studies indicate that the effects of this alcohol exposure are not global in nature but seem to affect certain areas more than others in both the neuropsychological and neuroanatomical arenas. Continuing studies are focusing on the relationship between neuropsychological and neuroanatomical data and hopefully will result in a clearer picture of the strengths and weaknesses of people with a history of heavy prenatal alcohol exposure, thereby allowing researchers and clinicians to develop more targeted and effective intervention approaches.

## Footnotes

1 The studies in animals, primarily rodents, were conducted shortly after birth, a period that corresponds to the third trimester of gestation in humans with respect to brain development.
